# Use of a Single Wearable Sensor to Evaluate the Effects of Gait and Pelvis Asymmetries on the Components of the Timed Up and Go Test, in Persons with Unilateral Lower Limb Amputation

**DOI:** 10.3390/s22010095

**Published:** 2021-12-24

**Authors:** Maria Stella Valle, Antonino Casabona, Ilenia Sapienza, Luca Laudani, Alessandro Vagnini, Sara Lanza, Matteo Cioni

**Affiliations:** 1 Laboratory of Neuro-Biomechanics, Department of Biomedical and Biotechnological Sciences, University of Catania, 95123 Catania, Italy; casabona@unict.it (A.C.); mcioni@unict.it (M.C.); 2 Section of Physiology, Department of Biomedical and Biotechnological Sciences, University of Catania, 95123 Catania, Italy; 3 Residency Program of Physical Medicine and Rehabilitation, Department of Biomedical and Biotechnological Sciences, University of Catania, 95123 Catania, Italy; ile.sapienz@gmail.com; 4 Department of Physical Medicine and Rehabilitation, Regina Margherita Hospital, 97013 Comiso, Italy; dott.saralanza@gmail.com; 5 Faculty of Sports, Campus de los Jerónimos, UCAM Catholic University of Murcia, 30107 Guadalupe, Spain; llaudani@ucam.edu; 6 BTS Bioengineering, 20024 Garbagnate Milanese, Italy; alessandro.vagnini@btsbioengineering.com; 7 Gait and Posture Analysis Laboratory, Policlinico Vittorio Emanuele-San Marco, University Hospital, 95123 Catania, Italy

**Keywords:** sensory–motor gait disorders, limb prosthesis, spatial–temporal analysis, kinematics, symmetry index

## Abstract

The Timed Up and Go (TUG) test quantifies physical mobility by measuring the total performance time. In this study, we quantified the single TUG subcomponents and, for the first time, explored the effects of gait cycle and pelvis asymmetries on them. Transfemoral (TF) and transtibial (TT) amputees were compared with a control group. A single wearable inertial sensor, applied to the back, captured kinematic data from the body and pelvis during the 10-m walk test and the TUG test. From these data, two categories of symmetry indexes (SI) were computed: One SI captured the differences between the antero-posterior accelerations of the two sides during the gait cycle, while another set of SI quantified the symmetry over the three-dimensional pelvis motions. Moreover, the total time of the TUG test, the time of each subcomponent, and the velocity of the turning subcomponents were measured. Only the TF amputees showed significant reductions in each SI category when compared to the controls. During the TUG test, the TF group showed a longer duration and velocity reduction mainly over the turning subtasks. However, for all the amputees there were significant correlations between the level of asymmetries and the velocity during the turning tasks. Overall, gait cycle and pelvis asymmetries had a specific detrimental effect on the turning performance instead of on linear walking.

## 1. Introduction

Motor deficit due to unilateral amputation of a lower limb can lead to functional limitations over almost all the activities of daily living. The presence of a prosthesis can partially restore being physically autonomous, also permitting an aesthetic recovery of an amputee’s integrity. All these factors are important in terms of personal image and quality of life, as they facilitate the patient’s social and work integration.

One of the consequences related to the use of prostheses is the appearance of asymmetries between the prosthetic and intact sides concerning kinematic and/or kinetic parameters of the gait cycle, such as the step length or velocity, or parameters related to the mobility of body segments, such as the pelvis or the trunk [1,2,3,4]. Local body asymmetries lead to a shift in the body’s center of gravity that can cause further detrimental effects; however, in many cases, asymmetries produce compensatory strategies that improve postural stability [5,6,7,8].

The effects of asymmetries in amputees have typically been studied during linear walking and, as reported by Devan et al. [9], most of the movement asymmetry studies in lower limb amputation have focused on local gait features, such as weight transfer during stance, generation of ground reaction forces, step time, and step length. On the contrary, the asymmetries concerning trunk and pelvic segments have received less attention, with the consequences of neglecting the effects of asymmetries on the mobility of daily living motor tasks, such as standing up, sitting down, or walking along a curved path.

In the current study, we chose to use the Timed Up and Go (TUG) test to quantify the physical mobility of a sample of subjects with lower limb amputation and evaluate the effects of both gait cycle and pelvic asymmetries among the subcomponents that are included in the test. In fact, the test consists of a sequence of natural actions that, starting from the sitting position, includes the following steps: standing up from a chair, walking 3 m forward, turning around an obstacle, walking back for 3 m, turn pivoting on one foot, and sitting down again. This test was introduced by Podsiadlo and Richardson [10] in order to permit the evaluation of the global mobility of frail older people adopting a simple and short duration test. In fact, the TUG test is frequently used not only for the assessment of physical mobility in the elderly population [11], but also in pathological contexts, such as Parkinson’s disease [12], muscular dystrophy [13], and stroke [14]. The extensive experience gained in the use of the TUG test in subjects with motor difficulties provides a rationale for its use to evaluate mobility also in lower limb amputees, paying specific attention to the different motor tasks included in the test. The TUG test was validated in people with a lower limb amputation [15,16], showing significant increase in the total time required to travel the entire path, when compared with the times measured in non-amputees [15,16,17,18,19].

Typically, the TUG test is performed using a simple stopwatch, but any errors due to operator reaction times can increase the variability of the measurement. A big leap forward for clinical and basic research took place when the use of wearable inertial measurement units (IMU) measured the TUG test (iTUG). The easy use of the wearable sensors and the capacity to provide spatiotemporal parameters make these devices suitable for capturing timing and kinematic data from both the entire path and from each TUG subcomponent [20]. As far as we know, only Clemens et al. [21] implemented a protocol to parameterize each subcomponent of the TUG test in people with a lower limb amputation, by using a mobile iPad application. These authors showed significant differences among the iTUG components, comparing transtibial (TT) with transfemoral (TF) amputees, especially for the mid-turning task. With respect to the descriptive information from the study of these authors, in the current work we used the TUG test, instrumented by a single wearable sensor, to add insights into the effects of the gait and pelvis asymmetries on the physical performance of the subcomponents of the TUG test, in TF and TT amputees.

## 2. Methods

### 2.1. Ethical Statement

This study was conducted in accordance with the Declaration of Helsinki Ethical Principles and Good Clinical Practices and was approved by the local ethics committee of Catania University Hospital “Policlinico Vittorio Emanuele-San Marco” (BIOART Project, n° 72/2019/PO). Participation was voluntary and all participants read and signed an informed written consent before starting the study.

### 2.2. Participants

Fourteen people with unilateral lower amputation were contacted and invited by the Physical Medicine and Rehabilitation Section of Regina Margherita Hospital (Comiso, Italy), to participate in the study. The inclusion criteria were: ≥18 years old, community-dwelling, unilateral leg or thigh amputation at any anatomical level, daily use of a prosthesis, and independent walking. The exclusion criteria were: bilateral amputation, fitting problems of the prosthesis, uncontrolled risk factors for cardiovascular disease, and presence of cognitive disorders. Among the selected subjects, one despondent and apparently inconsolable patient refused to participate as he did not perceive the study as relevant and 3 were unable to attend the health center. Therefore, 10 male amputees, classified by the evaluation practitioners at K3 or K4 level, according to the Medicare Functional Classification [22], were enrolled in the study.

Based on the level of amputation, the participants were divided into 2 groups, 5 belonging to the transfemoral (TF) group and 5 to the transtibial (TT) group. A third group of 5 healthy volunteers (CTRL) was recruited as the control. All the participants included in the three groups were male, with non-significant statistical differences for age, weight, and height (Table 1). The anthropometric and clinical data for each person with a lower limb amputation are reported in Table 2.

### 2.3. Experimental Procedures

All participants, wearing comfortable clothes in order to ensure adequate mobility, received standardized verbal and visual instructions and explanations about the experimental set-up and protocols so that equipment and rules were the same for everyone. As the motor tasks belonged to routine activities of daily life, the training regimen before recording was not considered necessary. The amputees wore their prostheses during the entire recording period of the procedures.

To assess balance ability, gait cycle, and pelvis symmetries, as well as physical mobility, the following procedures were used:

Test 1: Balance ability and risk of falling (Italian Version of Berg Balance Scale (BBS-it) [23]). The static and dynamic balance abilities and possible risk of falling for the amputees were assessed using the BBS-it. This is among the most used scales for balance evaluation in the rehabilitation field and its validity and reliability have also been well documented in persons with lower limb amputation [24]. Participants were required to perform a series of predetermined 14 tasks (14) of varying difficulty, scored from 0 (unable) to 4 (independent), so that the total score ranged from 0 to 56. This procedure took approximately 25 min to complete.

Test 2: 10-m walk test. The path was identified with a line marked with adhesive tape at the beginning and at the end of the 10 m. All participants were instructed to walk the path three times at a self-selected comfortable velocity. This protocol took approximately 10 min to complete, with an inter-trial rest interval of 2 min. We collected at least 24 gait cycles for each participant. From the total number of trials, we selected those that presented comparable measures of the walking velocity over the three groups of participants. For this purpose, over the total trials (3 for each subject), we identified the ranges of velocity values measured in the participant belonging to each group. The range of the TF amputees showed the lowest velocity values over the groups (0.7–1.0 m/s), the CTRL group showed a highest range of velocity values (0.9–1.2 m/s), while the TT amputees group exhibited an intermediate range of velocity values (0.8–1.1 m/s). For the CTRL and TT groups, we eliminated those trials with values out of the TF group range and verified that not significant differences occurred between each pair of groups. To evaluate the asymmetries of the gait cycle and pelvis, the following kinematic parameters were extrapolated for each cycle: body acceleration when the gait cycle was executed by left or right lower limb and pelvis angular displacements over the three planes of space.

**Figure 1:**
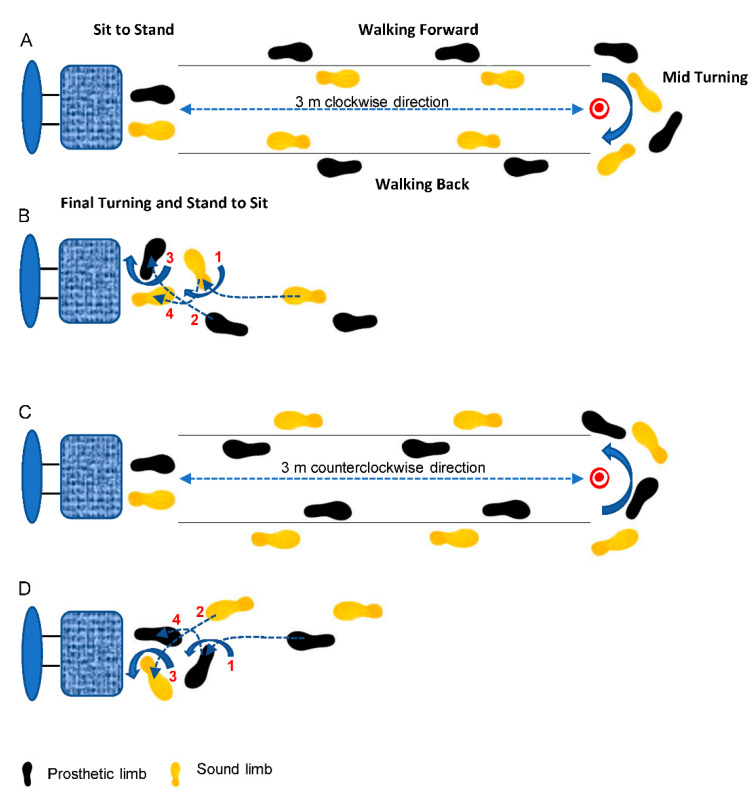
Top view of typical path of 3-m long for the TUG test. The test was performed in clockwise (**A**,**B**) and counterclockwise (**C**,**D**) directions. The single subcomponents are indicated in the panels (**A**,**B**). Four basic phases are performed to accomplish the final turning, before sitting down (**B**,**D**): (1) the body rotated of about 90° around the inner limb, (2) allowing the swing of the outer limb; (3) the body rotated of another 90° around the outer limb, (4) allowing the swing of the inner limb and the completion of 180° body rotation.

Test 3: TUG test (Figure 1). A 3-m-long rubber mat covered the floor and a chair without armrests was correctly placed at the beginning of the path. All participants executed the following sequence of simple tasks: stand up from the chair (sit to stand), walk along a straight line for 3 m (walking forward), turn 180° around a pin 18-cm high (m id-turning), walk back to the chair (walking back), turn for sitting (final turning), and sit down again (stand to sit). All participants were asked to walk at their own normal pace. The test was repeated six times, three times walking clockwise (Figure 1A,B) and the remaining three times counterclockwise (Figure 1C,D), to catch turning with internal and external prosthetic limb, respectively. The median values over the three clockwise and the three counterclockwise turns were used for the subsequent analyses. This protocol took approximately 15 min to complete, with an inter-trial rest interval of 2 min.

### 2.4. Data Collection and Processing

For tests 2 and 3, we used a commercial wearable inertial sensor (G-Sensor, BTS Bioengineering, Garbagnate Milanese, Italy) with dimensions of 70 × 40 × 18 mm, applied over the skin of the second lumbar vertebra. The sensor is composed of a triaxial accelerometer 16 bit/axes (sensor range, ±2 g), a triaxial magnetometer 13 bit (±1200 μT), and a triaxial gyroscope 16 bit/axes (sensor range, ±2000°/s). The signals were sampled with a frequency of 100 Hz and transmitted via Bluetooth to a laptop computer for acquisition and processing using a dedicated software package (BTS^®^ G-Studio, BTS Bioengineering, Garbagnate Milanese, Italy). For test 3, the subcomponents were identified using the criteria described by Negrini et al. [25]. Then, the following movement parameters were considered: total time duration of the iTUG test, duration of the single subcomponents, average velocities of mid- and final turning.

We used the 10-m walk test (test 2) to estimate gait cycle and pelvis symmetries, instead of the TUG test, because the former test provided less variability in the waveform average than the TUG test, which involved the alternation of short linear with curved walking.

Gait cycle symmetry estimation can be obtained by several mathematical tools on the basis of the differences in single measures between the two body sides, such as velocity, joint torque, or based on a more global estimation of the symmetry using correlation analysis on data captured over the entire left and right gait cycle (for a comprehensive review see [26]). In this study the correlation method was used to compute two symmetry measures: a symmetry index (SI) for total gait cycle (SI_gait_) and a SI for pelvis movements (SI_pelvis_).

**Figure 2:**
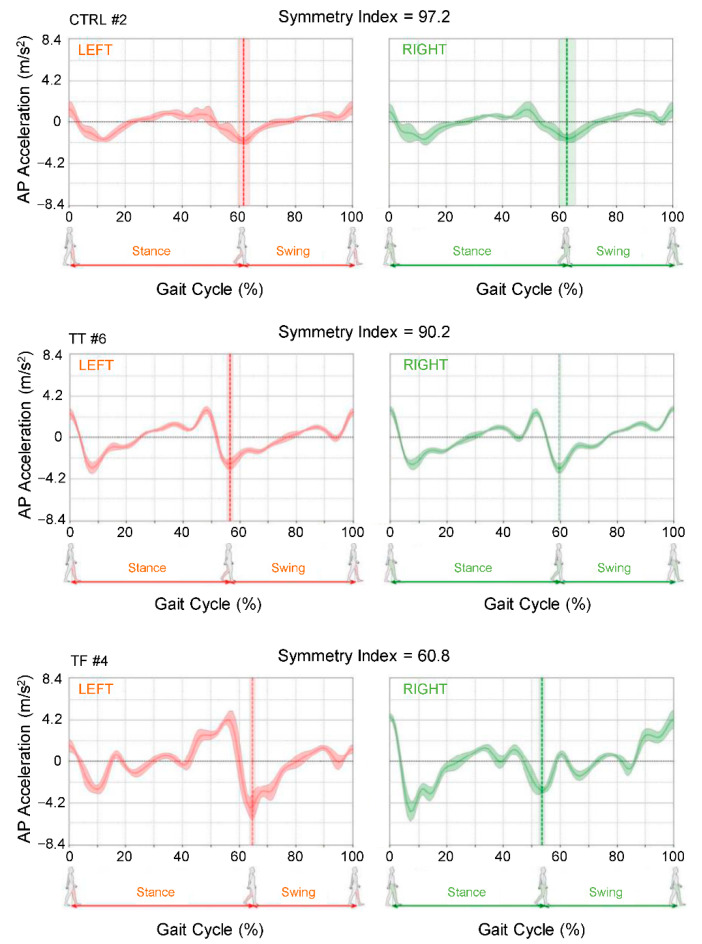
Representative examples of anterior–posterior (AP) body acceleration during gait cycle performed on the left and right sides by one control participant (CTRL), one transtibial amputee (TT), and one transfemoral amputee (TF). The waveforms represent the means and the standard deviations of AP accelerations from the total number of cycles during the 10-m walk test. The linear correlation between the left and the right AP accelerations, estimated by the Pearson correlation coefficient, was the basis for the gait cycle symmetry index (see Section 2 for details).

To estimate the SI_gait_, we correlated anterior–posterior (AP) body acceleration signals detected when the gait cycle was performed by the left or right lower limb (Figure 2). The SI_gait_ was obtained starting from the AP acceleration signal provided by the sensor as follows:

1. AP acceleration signal relative to the left gait cycles has been extracted from the whole acceleration signals;
2. AP acceleration signal relative to the right gait cycles has been extracted from the whole acceleration signals;
3. Mean normalized AP acceleration signal of the left gait cycles has been computed (acceleration signals on the left panels in Figure 2);
4. Mean normalized AP acceleration signal of the right gait cycles has been computed (acceleration signals on the right panels in Figure 2);
5. Compute Pearson’s correlation coefficients (r) between 3 and 4;
6. The SI_gait_ is obtained remapping the values of r, ranging from −1 to 1, between 0 and 100 with the following formula: SI_gait_ = (r + 1) × 100/2.

**Figure 3:**
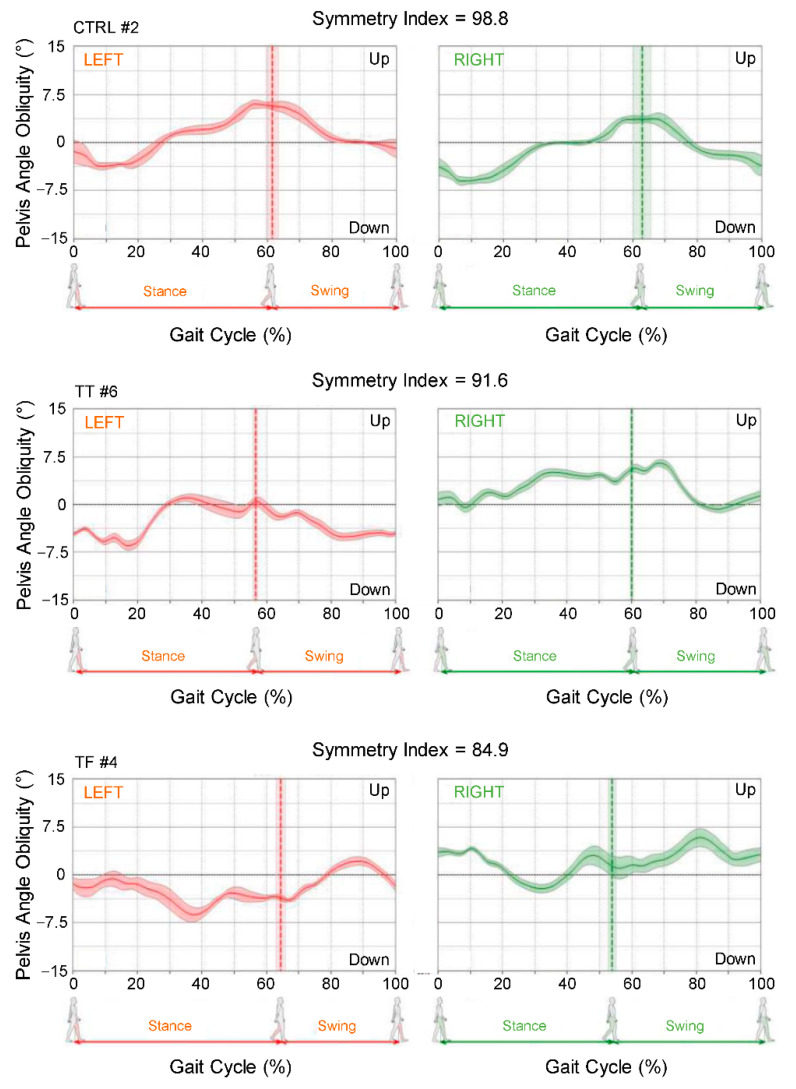
Representative examples of pelvis angle obliquity during gait cycle performed on the left and right sides by one control participant (CTRL), one transtibial amputee (TT) and one transfemoral amputee (TF). The waveforms represent the means and the standard deviations of pelvis angle obliquity from the total number of cycles during the 10-m walk test. The linear correlation between the left and the right pelvis angle obliquity, estimated by the Pearson correlation coefficient, was the basis for the gait cycle symmetry index. (See Section 2 for details).

Instead, the SI_pelvis_ was obtained from the correlation between the measures of the pelvic angles, when the gait cycle occurred on the left and right sides (Figure 3). Pelvic angular displacements have been computed by the software G-Studio (BTS Bioengineering, Milano) processing the rotational angles provided by the sensor (i.e., roll, pitch, and yaw) to obtain Cardan angles referred to a global reference system. Each SI_pelvis_ was obtained starting from the pelvic angle movement in the plane as follows:

1. Pelvic angle signal relative to the left gait cycles has been extracted from the whole pelvic angle signals;
2. Pelvic angle signal relative to the right gait cycles has been extracted from the whole pelvic angle signals;
3. Mean normalized pelvic angles signal of the left gait cycles has been computed (changes in amplitude of pelvic angle on the left panels in Figure 3);
4. Mean normalized pelvic angle signal of the right gait cycles has been computed (changes in amplitude of pelvic angle on the right panels in Figure 3);
5. Compute Pearson’s correlation coefficients (r) between 3 and 4
6. The SI_pelvis_ is obtained remapping the values of r, ranging from −1 to 1, between 0 and 100 with the following formula: SI_pelvis_ = (r + 1) × 100/2

In the case of the pelvis, a set of SI_pelvis_ was computed for the movements in the sagittal, frontal and transverse planes, obtaining tilt, obliquity and rotation SI_pelvis_, respectively.

On these bases, the larger value of symmetry index, the more similar body acceleration during gait cycle (SI_gait_) or pelvis angular displacements (SI_pelvis_) between the two sides will be.

### 2.5. Statistical Analysis

As each group included a small sample, we preliminarily analyzed data for normality distribution using Shapiro–Wilk’s test. The assumption of normality was not met for most of the parameters; therefore, nonparametric statistics were applied. Moreover, considering the small and poorly distributed samples, the significance level (*p* value) was computed based on the exact significance test. Finally, to determine the strength of the results, the magnitude of the effect was evaluated by epsilon-squared (ε^2^), as it is considered a less biased effect size estimator compared to others [27].

The outcomes for each group are represented as median, interquartile range and the minimum and maximum values. The mean and the standard deviation are reported when required. The Kruskal–Wallis *H*-statistic was performed to compare the three groups for each SI and TUG parameter, with Dunn’s test used for the post hoc analysis. To evaluate the differences between inner vs outer walking in each amputee group, the Wilcoxon Signed Rank test was adopted. Finally, Spearman’s test was used to explore the relationship between SI and TUG parameters in the amputees. Separate correlations were computed with respect to the limb prosthetic position during the TUG test. The strength of the linear correlation was assessed by Spearman’s Rho coefficient (r_s_).

For all the statistical comparisons, the alpha level of significance was set at 0.05.

## 3. Results

### 3.1. Gait and Pelvis Symmetry Evaluations

**Figure 4:**
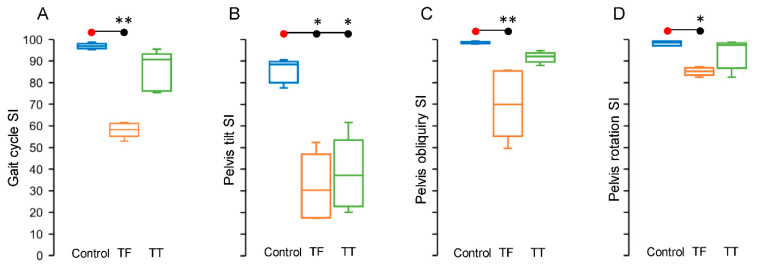
Box and whisker plots reporting the median value (horizontal line within the box) and the variability as interquartile range (vertical length of the box) and as the highest and the lowest values (lines above and below the box) of symmetry indexes of gait cycle (**A**), pelvis tilt (**B**), pelvis obliquity (**C**), and pelvis rotation (**D**), for each group. In all subplots, the horizontal lines with asterisks indicate statistically significant differences (* *p* < 0.05; ** *p* < 0.01). Abbreviations and symbols: SI = symmetry index; TF = transfemoral group (orange boxes); TT = transtibial group (green boxes).

The indices of gait cycle and pelvis symmetries were lower in the participants with amputation with respect to the individuals of the CTRL group for all the evaluations (Figure 4A–D).

Participants with TF amputations showed the lowest values of SI, compared to CTRL and TF groups, for the SI_gait_ (Figure 4A; median [interquartile range]: CTRL, 96.9 [2.2]; TF, 58.3 [6]; TT, 90.6 [17]), the pelvis tilt SI (Figure 4B; CTRL, 88.5 [9.7]; TF, 30.4 [29.4]; TT, 37.2 [30.8]), the pelvis obliquity SI (Figure 4C; CTRL, 98.3 [0.8]; TF, 69.9 [30.3]; TT, 92.1 [4.2]) and pelvis rotation SI (Figure 4D; CTRL, 98.6 [1.9]; TF, 85.2 [3.3]; TT, 97.4 [11.6]).

There were significant differences across the groups for SI evaluated for gait cycle (*H*_2_ = 12.02, *p* < 0.001, ε^2^= 0.86), pelvis tilt (*H*_2_ = 9.62, *p* = 0.002, ε^2^= 0.69), pelvis obliquity (*H*_2_ = 12.5, *p* < 0.001, ε^2^= 0.89), and pelvis rotation (*H*_2_ = 7.74, *p* = 0.012, ε^2^= 0.55).

The post hoc comparisons showed that this is the result of a significant difference observed only between the CTRL and TF groups for SI_gait_ (Figure 4A; *p* = 0.002), pelvis obliquity SI (Figure 4C; *p* = 0.002) and pelvis rotation SI (Figure 4D; *p* = 0.012), while in the case of pelvis tilt SI, there were significant differences between the CTRL and TF groups (Figure 4B; *p* = 0.011) and between the CTRL and TT groups (Figure 4B; *p* = 0.049). No significant differences were observed between TF and TT groups for all SI evaluations.

### 3.2. Timed Up and Go Component Results

**Figure 5:**
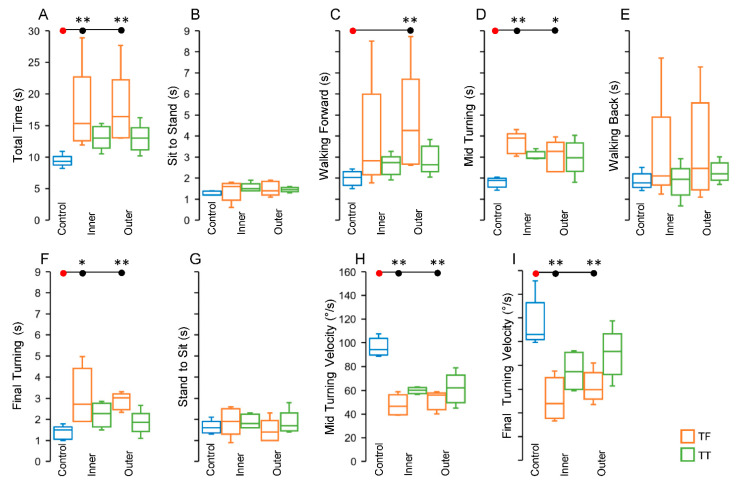
Box and whisker plots reporting median and variability (as described in Figure 4) of parameters measured during the Timed Up and Go test: Total time (**A**), sit to stand (**B**), walking forward (**C**), mid-turning (**D**), walking back (**E**), final turning (**F**), (**G**) stand to sit, (**H**) mid-turning velocity, (**I**) final turning velocity. Abbreviations as in Figure 4. Symbols: * *p* < 0.05; ** *p* < 0.01.

The TUG total time and the measures obtained in each TUG component among the CTRL, TF, and TT groups were statistically analyzed by two sets of comparisons considering whether the prosthetic limb was in the inner or outer position during mid-turning. Thus, in each box plot of Figure 5, the data regarding the CTRL group (blue box), obtained with the TUG test performed in a clockwise direction, were compared with the results from the TF (orange box) and TT (green box) groups with separate analysis for the inner or outer prosthetic limb position. To facilitate a comparison between the results of the TUG test obtained in this study and those reported by other studies, in Table 3 we provide numerical data of the TUG test results expressed as mean and standard deviation.

The results of the non-parametric statistical analysis for the TUG test are summarized in Table 4. In the following paragraphs the time measures are expressed in seconds, in cases of linear iTUG subcomponents, and the velocity measures in degree per seconds, in cases of Mid and Final Turning.

Statistical differences among the three groups were detected for the TUG total time (Figure 5A) both when the prosthetic limb was in the inner position and when it was in the outer position (see Table 4 for the results of Kruskal–Wallis test). The TF group showed the highest value of TUG total time (Table 4 and Figure 4A; median [interquartile range]: CTRL, 9.3 [1.35]; TF inner, 15.3 [10.1]; TT inner, 13 [3.4]; TF outer, 16.4 [9.15]; TT outer, 13 [3.5]), and the only significant difference among the post hoc pairwise comparisons was found between the CTRL and the TF groups (inner, *p* = 0.008; outer, *p* = 0.004; see Table 4 for more details).

The values observed for the TUG total time depended mainly on the changes in time and velocity during mid-turning (Figure 5D,H) and final turning (Figure 5F,I). In fact, as reported in Table 4 (Kruskal–Wallis test), the pattern of statistical differences observed for the total time, with the significant differences focused on the comparison between CTRL and TF groups, was replicated by the measures revealed during the mid-turning time (Figure 5D; CTRL, 1.89 [0.42]; TF inner, 3.9 [0.94]; TT inner, 2.95 [0.32]; TF outer, 3.27 [1.41]; TT outer, 2.97 [1.35]), the final turning time (Figure 5F; CTRL, 1.5 [0.6]; TF inner, 2.72 [2.51]; TT inner, 2.27 [1.12]; TF outer, 3.02 [0.76]; TT outer, 1.86 [0.86]), the mid-turning velocity (Figure 5H; CTRL, 94.1 [14.1]; TF inner, 46.7 [16.9]; TT inner, 60.2 [5.3]; TF outer, 56 [13.9]; TT outer, 62.1 [23]) and the final turning velocity (Figure 5I; CTRL, 106 [31.15]; TF inner, 47.9 [34.35]; TT inner, 74.5 [30.75]; TF outer, 59.6 [21.85]; TT outer, 91.7 [34.35]).

No significant differences among groups, for both inner and outer prosthetic limb positions, were observed for the other TUG components, except for walking forward (Figure 5C), which showed significant differences for groups when the prosthetic limb was in the outer position, with pairwise significant differences between CTRL and TF outer (*p* = 0.007; see Table 4 for more details).

The comparison between inner and outer conditions as repeated measures within TF and TT groups was conducted by the Wilcoxon signed rank test and revealed that no statistically significant differences occurred for all the measured TUG parameters (see details in Table 4).

For all the statistically significant results, there was a good magnitude of the effect size, with ε^2^ ranging from 0.59 to 0.83 for the data related to the condition with the prosthetic limb in the inner position, and from 0.53 to 0.74 for the data related to the condition with the prosthetic limb in the outer position.

### 3.3. Correlations between Symmetry Indices and TUG Test Measures

**Figure 6:**
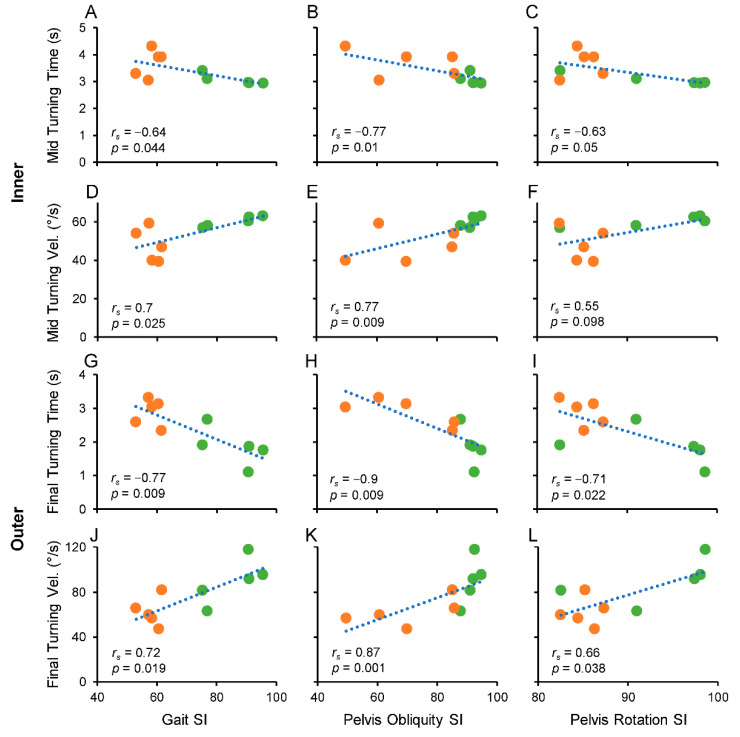
Spearman’s correlation analysis between symmetry index and mid- and final turning time and velocity. Symmetry indexes of gait cycle, pelvis obliquity, and pelvis rotation were correlated with mid-turning time and velocity, when the amputated limb was in inner position (**A**–**F**), and with final turning time and velocity, when the amputated limb was in outer position (**G**–**L**). Abbreviation and symbol: *r_s_,* Spearman’s Rho coefficient; *p*, level of significance; orange circles, TF; green circles, TT.

The correlations between the changes in SI parameters and the measures of TUG subcomponents are reported in Table 5. As can be seen from Table 5, almost all the statistically significant correlations (*p* < 0.05; in bold in the Table 5) are those where the SI of the gait cycle, pelvis obliquity, and pelvis rotation are related to the mid-turning time and velocity when the prosthetic limb was in the inner position during mid-turning (Figure 6A–F) and to the final turning time and velocity when the prosthetic limb was in the outer position during mid-turning (Figure 6G–L).

The level of correlation, estimated by the Spearman coefficient (*r_s_*), ranged from 0.64 to 0.77 for the correlations associated with mid-turning, and from 0.66 to 0.9 for the correlations associated with final turning.

The plots in Figure 6 show that as SI increased, the turning time decreased (Figure 6A–C,G–I) or the turning velocity increased (Figure 6D–F,J–L), indicating that gait cycle and pelvis asymmetries associated with amputation influenced the performance during rotation components of the TUG test more than the performance during the execution of linear TUG components.

Moreover, the two amputee groups tended to cluster into two separate groups, with the TT group (green circle) fitting the linear model better than TF group (orange circle) in the case of the correlations associated with mid-turning time or velocity (Figure 6A–F).

Finally, the BBS-it test showed significant correlations with the measures of TUG subcomponents mainly during mid- and final turning, for both the inner and outer positions of the prosthetic limb (last two columns on the right, in Table 5). The velocity of turning increased as balance increased.

## 4. Discussion

The basic result reported here is that subjects with an amputation took more time to complete the TUG test and showed lower levels of gait and pelvis SI than non-amputee volunteers. However, almost all the statistical differences between amputees and healthy people were focused on the TF group.

The TUG test duration in the case of amputees depended mainly on the slowdown observed during mid- and final turning. Moreover, the velocity reduction during the turning components of the TUG test was well correlated with the reduction in gait cycle and pelvis symmetries observed in the amputees. In particular, the association between reduction in velocity and reduction in gait cycle and pelvis symmetries occurred when the prosthetic limb was in the inner position during mid-turning and in the outer position during final turning.

### 4.1. The Turning Components of the TUG Test Represent the Most Demanding Tasks for the Amputees

The results of the current study provide insights into the possible causes for the specific difficulties showed by the amputees in facing the turning task, suggesting that gait cycle and pelvis asymmetries may contribute to differentiate the level of mobility between linear and curved walking.

To our knowledge, only Clemens et al. [21] have used the TUG test to explore the performance over the single task components. Although the number of participants with lower limb prostheses in the study of Clemens et al. [21] was much larger (a total of 118 participants) than the sample size used in the current work, their main result supports our main finding as these authors indicate that, among the tasks included in the TUG test, the turning components have the greatest impact in the level of mobility of subjects with lower limb amputation. Although the high number of participants in the study of Clemens at al. [21], the mean time duration and the variability observed by these authors between TT and TF amputees, during the mid-turning, were comparable with the scores recorded in the current study: for the TT amputees, 2.73 s ± 0.65 in Clements et al. [21], 3.05 s ± 0.5 in the current study (average between inner and outer limbs); for the TF amputees, 3.53 s ± 1.2 in Clements et al. [21], and 3.4 s ± 0.6 in the current study (average between inner and outer limbs). However, the large difference in the sample size between the two studies determined significant statistical differences between TT and TF amputees for all the TUG subcomponents in the study of Clements et al. [21], with some subcomponents, especially for walking forward, showing large duration and variability in the current study. This reflects the different time duration in the TUG total time between the two works: for the TT amputees, 10.04 s ± 2.3 in Clements et al. [21], 13 s ± 2.05 in the current study (average between inner and outer limbs); for the TF amputees, 12.77 s ± 5.04 in Clements et al. [21], 17.3 s ± 6.4 in the current study (average between inner and outer limbs).

Several authors showed evidence that asymmetries in subjects with lower limb amputations might be the result of motor adaptations to accomplish functional compensations such as, facilitating lower-leg trajectory [8], foot clearance during the swing phase of gait [7], gait stability [6] and functional step length and symmetrical thigh inclinations [5]. Typically, these adaptations are related to walking in a straight line, therefore, the appearance of asymmetry may be functional to a linear path, but it may not be suitable for the path along a curve, impairing the turning task.

### 4.2. Velocity Decrease and Asymmetry Effects When Amputees Face Turning Points: Biomechanical and Neuronal Considerations

In an attempt to provide an explanation for the changes in the kinematics and for the effects of gait and pelvis asymmetries on mid- and final turning, in the following section we consider the basic biomechanics and neuronal processes underlying a successful execution of a turning task.

#### 4.2.1. Biomechanical Considerations

In non-amputees, the process of turning starts before the change of direction, with forward motion deceleration followed by body rotation and the movement toward the new direction [28,29].

The two turning components of the TUG test are performed on the basis of different biomechanical strategies. Mid-turning can be included in the category of step turning, since the body is stepping along an arc of 180°, while final turning can be considered a form of spin turning, as the step is stopped, and the body axis rotates about 180° [28,29,30,31]. During step turning, the body center of mass typically moves over the mediolateral axis, shifting in the direction of the turn, at the boundary of the base of support. As the velocity of turning decreases, the center of mass slightly shifts between the two feet, within the base of support [32]. This process provides a straightforward explanation for the reduced velocity observed during mid-turning in the subjects with a lower limb amputation: the increased risk to perturb body stability forced the amputees to reduce their velocity to place the center of mass in a more reliable position to maintain stability during the turning trajectory.

This time compensation could be particularly effective in cases of TF amputees who show more significant reductions in turning velocity than TT amputees when compared with the CTRL group. However, when mid-turning was negotiated with the prosthetic limb in the inner position, both amputee groups showed a reduction in velocity as gait cycle and pelvis obliquity asymmetries increased. This specific influence of pelvis obliquity asymmetry makes sense if we consider that the medial-lateral axis, along which the center of mass moves during mid-turning, is the same axis along which the pelvis oscillates when its obliquity changes. Moreover, the internal shift of the body center of mass produces a greater load on the inner limb, exacerbating the negative effects of pelvis asymmetry over the transverse plane.

Contrary to what we observed in mid- and final turning, there were no significant differences in timing and no influences of asymmetries in the linear components of the TUG test. The different responses observed in linear and turning walks may depend on the intrinsic lower stability characterizing turning with respect to walking in a straight line [33]. In subjects with a lower limb amputation these differences in stability are accentuated, since they are more sensitive to perturbations occurring along the medial-lateral axis, mainly present during turning, than perturbations that affect the anterior-posterior axis, mainly occurring during linear walking [34,35]. The parallel reduction in the BBS-it test and the velocity during turning tasks, observed in our experiments, confirms the tendency of amputees to be less stable in curved than in straight paths.

The reduction in velocity observed during mid-turning for TF amputees and the correlations with the changes in symmetry were also observed in final turning, before sitting down. The large curved path of step turning (mid-turning) offers greater stability compared to the spin turn (final turning) that requires the body to rotate around the vertical axis [28,29,31]. Although we did not observe differences in velocity reduction between the two types of turning, the enhanced instability attributed to final turning may explain the greater negative influences of asymmetries observed for final turning compared to mid-turning. In fact, the levels of correlation detected during final turning where higher than those observed during mid-turning, with significant correlations not only for gait cycle and pelvis obliquity asymmetries but also for the asymmetries associated with pelvis rotation (see correlation analyses illustrated in Figure 6G–L).

Another difference that we observed between the two turning tasks was that the influence of decreased symmetries at final turning occurred when the TUG test was executed with the prosthetic limb in the outer position. The transition from turning and stand to sit, illustrated in Figure 1B,D, provides a possible explanation for the prevalent influence of asymmetries when final turning is performed by the prosthetic limb in the outer position. This transition occurs accomplishing a 180° body rotation through two semi-rotations: the first semi-rotation of the body, of about 90°, occurs around the inner limb (point 1 in Figure 1B,D), allowing the swing of the outer limb (point 2 in the Figure 1B,D); the second semi-rotation, of another 90°, takes place around the outer limb (point 3 in the Figure 1B,D), allowing the swing of the inner limb (point 4 in the Figure 1B,D), and the completion of the 180° rotation of the body. During the first semi-rotation, the static load on the inner limb is reduced due to the residual energy from the dynamics of the last step before starting final turning. Instead, the velocity reduction occurred when the step cycle stopped, producing an increase in static load on the outer limb during the second semi-rotation. Thus, the outer limb receives more load than the inner limb, becoming more susceptible to the effects of pelvis asymmetries.

#### 4.2.2. Neuronal Considerations

The best performance observed in amputees during linear compared to curved walking could depend on a specific need to adopt a predictive feedforward strategy in the curved walk, with respect to the straight line walk.

A critical factor for successful turning biomechanics is to plan trunk and pelvis rotations in advance, during the deceleration period preceding the turning phase [28,29,32,36]. A walking direction-dependent neuronal control was shown by Bauby and Kuo [37] and O’Connor and Kuo [38] who observed that reduced visual information destabilized more mediolateral than anteroposterior motions during walking. Since visual sensory feedback is essential for planning in advance the changes in walking direction, these authors concluded that linear walking relies more on the passive mechanical properties of the limbs, while for stabilizing mediolateral motion a significant central active control must be provided. As discussed in the previous section, amputees show greater difficulty in stabilizing perturbations that act along the mediolateral axis, with respect to those that act along the anterior-posterior axis [34,35]. Thus, a deficit in neuronal predictive control could be an explanation for the reduced turning performance in amputees, particularly for those with a TF amputation.

Difficulties in anticipatory strategy were observed in TT amputees when movements of the upper limbs perturbed upright standing [39] or lateral pushes perturbed linear walking [40]. Similar deficits were detected in older subjects who showed limitations in facing mid- and final turning during the TUG test [20] and difficulties in anticipatory adjustments after a later perturbation during gait initiation [41]. Similar to the condition of amputees, it can be assumed that also physical body variations associated with aging make predictive capacity more difficult to implement.

Planning a movement strategy in advance to face future biomechanical changes, the inside shift of the center of mass in the case of mid-turning, requires not only an exteroceptive sensory feedback, but also an accurate internal representation of the relationships between the mechanical state of the body and the contextual environment (predictive internal models; [42]). Similarly, during final turning, for successful rotation a proactive neuronal control in relation to the subsequent stand to sit task is necessary. Rebuilding an internal model of the body is one of the greatest difficulties for subjects with an amputation since the missing part and the prosthesis upset the biomechanical and temporal relationships between body segments. This issue was recently addressed by Saimpont et al. [19] using a motor imagery protocol applied to amputees performing 7-m walking and the TUG test. These authors found timing and accuracy discrepancies in amputees comparing the actual execution and the mental imagery of the TUG test or walking. It was suggested that these discrepancies were due to dysfunctions in the internal predictive models that need to be updated. The motor imagery of the total time of the TUG test tended to be slower than the real execution with respect to 7-m walking. However, in the study of Saimpont et al. [19], no parameter measurements were recorded for the TUG subcomponents.

In summary, the basic biomechanics and neuronal processes to accomplish the transition from linear to curved walking during the TUG test suggest that velocity reductions in TF amputees and the negative influence of gait cycle and pelvis asymmetries in both amputee groups, may be attributable, at least in part, to the difficulty of the amputees in updating forward internal models capable of preventing the effects of perturbations acting on the mediolateral axis of the body during turning tasks.

## 5. Conclusions and Practical Implications

The main message from the current study is that the idea of asymmetry occurring in amputee motions to compensate for the differences between the two limbs is not applicable to the various motor actions. Gait cycle and pelvis asymmetries are appropriate for balancing linear walking, but they can be detrimental for turning performance. Considering that predictive control is a specific and critical factor for the successful transition from linear to curved walking, we suggest reinforcing those rehabilitative protocols addressed to stimulating the sensory feedback from the residual limb segments so as to trigger an update of the internal body representation and the forward models. For example, considering that amputees have no neurological injury, using the appropriate accommodations, the use of a tool such as a balance board can be important to stimulate the proprioceptive afferences and produce learning of new postural skills, as was demonstrated for healthy individuals [43,44]. Moreover, recent studies have demonstrated that the use of new tools, such as motor imagery [19,45] and virtual reality [46], can provide an important support in training and monitoring the development and updating of internal models, in subjects with a lower limb amputation.

Finally, as the use of a single wearable sensor contributed significantly in this study to detecting measures from different motor contexts, similarly the use of these devices can improve the quality of rehabilitation protocols, making the measurements of demanding motor tasks easier and more reliable [47,48].

## 6. Limitations

The reduced sample size, made up of male participants, may represent only a portion of the population. This is a limitation to consider for the interpretation of the results reported in the current study. In addition, some characteristics, such as the type of prothesis or the time elapsed since amputation, are not homogenous over the amputees. However, we tried to adopt the most suitable statistical procedures to partially compensate for these limitations. We believe that the level of significance and the magnitude of the effect size should guarantee the reliability of the main results. Further studies including a larger sample of people with a lower limb amputation could reinforce the general character of these results.

## Figures and Tables

**Table 1:** Descriptive statistics (mean ± standard deviation and range) of the anthropometric data of all participants.

	Groups			*t* Test (*p*)		
	TT	TF	CTRL	CTRL vs. TT	CTRL vs. TF	TT vs. TF
Age (yrs)	50.6 ± 9.1 (41–64)	53.6 ± 14.9 (34–71)	49.6 ± 8.5 (43–63)	0.862	0.617	0.712
W (kg)	84.8 ± 15.6 (73–110)	81.2 ± 14.8 (71–106)	78.6 ± 7.2 (72–90)	0.444	0.734	0.719
H (cm)	172.8 ± 4.9 (165–178)	171.8 ± 8.8 (165–187)	174.4 ± 6.3 (164–181)	0.664	0.605	0.829

CTRL, control group; TF, transfemoral group; TT, transtibial group; W, weight; H, height; *p*, level of significance.

**Table 2:** Anthropometric and clinical data of the persons with lower limb amputation.

Subjects	Level	Age (yrs)	W (kg)	H (cm)	BMI	Onset (yrs)	Side	Cause	Falls (n)	BBS Score	Knee	Foot
#1	TF	50	106	187	30.3	3	Left	T	4	43	RK3	VF-LP
#2	TF	66	74	165	27.2	4	Right	V	0	52	PABHD	AM
#3	TF	71	71	171	24.3	5	Right	V	1	41	PABHD	AM
#4	TF	47	71	167	25.4	32	Right	T	2	50	P	VF
#5	TF	34	84	169	29.4	4	Right	T	2	52	PTK	SACH
#6	TT	41	73	165	26.8	3	Left	T	0	54		VF
#7	TT	55	90	172	30.4	20	Left	T	0	55		PF-XC
#8	TT	48	74	174	24.6	4	Left	T	3	56		VF-HS
#9	TT	45	110	178	34.7	30	Right	T	0	54		PF-XL
#10	TT	64	77	175	25.1	1	Left	V	0	40		1D1

TF, transfemoral; TT, transtibial; T, trauma; V, vascular; RK3, Rheo Knee 3 microprocessor; PABHD, polyfunctional automatic brake hydraulic device; P, polycentric; PTK, polycentric total knee 1900; VF-LP, Vari-Flex-LP; AM, articulated multi-axis: VF, Vari-Flex; VF-HS, Vari-Flex-Harmony; PF-XL, Pro-Flex XL; PF-XC, Pro-Flex XC; 1D1 = 1D1-Dynamic.

**Table 3:** Descriptive statistics (mean ± standard deviation) for temporal and velocity parameters over the TUG subcomponents, in the persons with lower limb amputation and non-amputated.

	Temporal Parameters (s)							Velocity Parameters (°/s)	
Groups	Total Time	Sit to Stand	Walking Forward	Mid Turning	Walking Back	Final Turning	Stand to Sit	Mid Turning	Final Turning
CTRL	9.4 ± 1.0	1.3 ± 0.1	2.0 ± 0.4	1.8 ± 0.2	1.9 ± 0.4	1.4 ± 0.3	1.6 ± 0.3	96.1 ± 7.7	115.0 ± 20.9
TF Inner	17.2 ± 6.8	1.4 ± 0.5	3.8 ± 2.7	3.7 ± 0.5	3.1 ± 2.6	3.1 ± 1.3	1.9 ± 0.7	47.7 ± 8.7	51.5 ± 17.8
TF Outer	17.4 ± 6.0	1.5 ± 0.3	4.6 ± 2.5	3.1 ± 0.7	3.3 ± 2.5	2.9 ± 0.6	1.5 ± 0.5	51.9 ± 7.8	62.1 ± 12.9
TT Inner	13.1 ± 1.9	1.6 ± 0.2	2.6 ± 0.5	3.1 ± 0.2	1.8 ± 0.8	2.2 ± 0.6	1.9 ± 0.3	60.0 ± 2.7	75.2 ± 15.4
TT Outer	12.9 ± 2.2	1.5 ± 0.1	2.9 ± 0.7	3.0 ± 0.8	2.3 ± 0.5	1.9 ± 0.6	1.8 ± 0.6	61.4 ± 12.7	89.8 ± 20.0

CTRL, control group; TF, transfemoral group; TT, transtibial group.

**Table 4:** Results of Kruskal–Wallis and Wilcoxon signed rank test analyses.

TUG Components	Kruskal-Wallis CTRL vs. TF vs. TT			Post Hoc (Dunn-Bonferroni)						Wilcoxon Signed Rank Test	
				CTRL vs. TF		CTRL vs. TT		TF vs. TT			
	*H*_2_	*p*	ε^2^	*z*	*p*	*z*	*p*	*z*	*p*	*z*	*p*
Prosthetic limb INNER										Transfemoral Inner vs. Outer	
Total time	9.57	**0.002**	0.68	8.5	**0.008**	−5.9	0.108	2.6	1	−0.272	0.938
Sit to stand	4.65	0.093	0.33							−0.271	0.875
Walking forward	5.04	0.075	0.36							−2.023	0.063
Mid-turning time	11.2	**<0.001**	0.80	9.4	**0.003**	−5.6	0.143	3.8	0.536	−1.483	0.188
Walking back	1.56	0.482	0.11							−0.135	1.000
Final turning time	8.31	**0.007**	0.59	8.0	**0.013**	−5.2	0.194	2.8	0.96	−0.135	1.000
Stand to sit	2.06	0.376	0.15							−1.219	0.313
Mid-turning velocity	11.58	**<0.001**	0.83	9.6	**0.002**	5.4	0.169	−4.2	0.413	−1.753	0.125
Final turning velocity	10.5	**<0.001**	0.75	9.0	**0.004**	6.0	0.102	−3.0	0.867	−1.753	0.125
Prosthetic limb OUTER										Transtibial Inner vs. Outer	
Total time	10.37	**<0.001**	0.74	9.0	**0.004**	5.4	0.165	3.6	0.602	−0.135	1.000
Sit to stand	3.61	0.167	0.26							−0.412	0.813
Walking forward	9.38	**0.009**	0.67	8.6	**0.007**	5.2	0.198	3.4	0.688	−0.405	0.813
Mid-turning time	7.35	**0.016**	0.53	7.0	**0.04**	6.2	0.085	0.8	1	−0.135	1.000
Walking back	1.72	0.448	0.12							−2.023	0.063
Final turning time	9.59	**0.002**	0.68	8.7	**0.006**	3.6	0.607	5.1	0.213	−0.674	0.625
Stand to sit	1.83	0.433	0.13							−0.404	0.812
Mid-turning velocity	9.98	**<0.001**	0.71	8.6	**0.007**	6.4	0.071	2.2	1	−0.134	1
Final-turning velocity	9.26	**0.003**	0.66	8.6	**0.007**	4.0	0.472	4.6	0.312	−0.943	0.437

CTRL, control group; TF, transfemoral group; TT, transtibial group; *H*_2_, Kruskal–Wallis statistic adjusted for ties; *p*, level of significance; ε^2^, epsilon squared effect size; *z,* Wilcoxon signed rank test statistic. Statistically significant differences are marked in bold.

**Table 5:** Results of Spearman’s correlation analysis.

TUG Components	Symmetry Index								BBS-it	
	Gait Cycle		Pelvis Tilt		Pelvis Obliquity		Pelvis Rotation			
	*r_s_*	*p*	*r_s_*	*p*	*r_s_*	*p*	*r_s_*	*p*	*r_s_*	*p*
Prosthetic limb INNER										
Total time	−0.44	0.199	0.01	0.973	−0.43	0.22	−0.10	0.776	−0.59	0.074
Sit to stand	0.10	0.789	0.54	0.111	0.12	0.738	0.40	0.258	−0.18	0.624
Walking forward	−0.47	0.174	−0.25	0.489	−0.38	0.276	−0.16	0.651	−0.45	0.197
Mid-turning time	−0.64	**0.044**	−0.32	0.374	−0.77	**0.01**	−0.63	**0.05**	−0.59	0.071
Walking back	−0.20	0.575	0.08	0.827	−0.61	0.063	−0.36	0.304	−0.75	**0.013**
Final turning time	−0.30	0.393	0.17	0.638	−0.16	0.65	0.28	0.434	−0.52	0.121
Stand to sit	−0.14	0.7	0.27	0.455	−0.35	0.327	−0.12	0.751	−0.28	0.426
Mid-turning velocity	0.70	**0.025**	0.37	0.293	0.77	**0.009**	0.55	0.098	0.66	**0.038**
Final turning velocity	0.55	0.098	−0.12	0.751	0.43	0.214	0.02	0.96	0.68	**0.03**
Prosthetic limb OUTER										
Total time	−0.65	**0.041**	−0.29	0.422	−0.67	**0.034**	−0.33	0.353	−0.60	0.068
Sit to stand	−0.13	0.724	−0.27	0.454	−0.01	0.973	0.06	0.867	0.21	0.569
Walking forward	−0.46	0.187	−0.03	0.934	−0.52	0.128	−0.21	0.556	−0.73	**0.018**
Mid-turning time	−0.12	0.738	0.14	0.7	−0.42	0.228	−0.28	0.434	−0.34	0.337
Walking back	−0.08	0.829	−0.48	0.162	−0.01	0.987	0.21	0.556	−0.45	0.197
Final turning time	−0.77	**0.009**	−0.07	0.855	−0.90	**<0.001**	−0.71	**0.022**	−0.70	**0.026**
Stand to sit	0.33	0.353	0.32	0.362	0.16	0.662	−0.04	0.907	0.41	0.238
Mid-turning velocity	0.36	0.31	0.24	0.511	0.58	0.082	0.18	0.627	0.67	**0.034**
Final turning velocity	0.72	**0.019**	0.06	0.881	0.87	**0.001**	0.66	**0.038**	0.71	**0.022**

*r_s_,* Spearman’s Rho coefficient; *p*, level of significance; BBS-it, Italian Version of Berg Balance Scale. Statistically significant values are shown in bold.

## Data Availability

The data that support the findings of this study are available on request from the corresponding author, M.S.V. The data are not publicly available due to the fact that they contain information that could compromise the privacy of research participants.
